# How collective comparisons emerge without individual comparisons of the options

**DOI:** 10.1098/rspb.2014.0737

**Published:** 2014-07-22

**Authors:** Elva J. H. Robinson, Ofer Feinerman, Nigel R. Franks

**Affiliations:** 1 School of Biological Sciences, Bristol University, Woodland Road, Bristol BS8 1UG, UK; 2 Department of Physics of Complex Systems, Weizmann Institute of Science, Rehovot, Israel

**Keywords:** decision-making, collective decisions, comparative evaluation, social insects, RFID, emigration

## Abstract

Collective decisions in animal groups emerge from the actions of individuals who are unlikely to have global information. Comparative assessment of options can be valuable in decision-making. Ant colonies are excellent collective decision-makers, for example when selecting a new nest-site. Here, we test the dependency of this cooperative process on comparisons conducted by individual ants. We presented ant colonies with a choice between new nests: one good and one poor. Using individually radio-tagged ants and an automated system of doors, we manipulated individual-level access to information: ants visiting the good nest were barred from visiting the poor one and vice versa. Thus, no ant could individually compare the available options. Despite this, colonies still emigrated quickly and accurately when comparisons were prevented. Individual-level rules facilitated this behavioural robustness: ants allowed to experience only the poor nest subsequently searched more. Intriguingly, some ants appeared particularly discriminating across emigrations under both treatments, suggesting they had stable, high nest acceptance thresholds. Overall, our results show how a colony of ants, as a cognitive entity, can compare two options that are not both accessible by any individual ant. Our findings illustrate a collective decision process that is robust to differences in individual access to information.

## 1. Introduction

Comparative assessment is a powerful tool for choosing between options, and it is widely implemented by decision-making animals, including insects, crabs, birds, bears and primates [1–7] and is even used by slime moulds [8]. However, comparative assessments can have drawbacks, including the emergence of ‘economically irrational’ behaviour [6–8] and the potential for cognitive overload [9–11].

House-hunting social insects are a model system for studying decision-making, demonstrating the ability to make effective choices in a range of choice contexts [12–15]. In these collective decision processes, there are two levels at which comparative assessment can be performed: individual and group. Ant colonies choosing between potential new nest-sites seem to be immune from both irrational behaviours [16,17] and cognitive overload [16,18], which would seem to indicate that comparative assessment does not play a role in collective decisions. At the individual-level, ants are capable of making comparative assessments [19], but their decision-making behaviour during emigration to a new nest-site can be explained without invoking comparison [20]. A simple threshold model in which ants either reject nests as being unsuitable and continue searching, or accept nests and recruit nest-mates to them, but do not directly compare available nests, reproduces observed collective decision behaviour [21]. In this study, we ask which individual-level behavioural rules are important for robust collective-scale decisions. Specifically, we ask whether the ability of individual ants to make comparative assessments of available options plays an essential role in collective decisions and, if not, whether the actions of partially informed individuals are consistent with the predictions of a simple threshold model of decision-making [21].

To test the role of individual comparisons in collective decision-making, we used radio-tagged ants and an automated system of doors, so that without physically removing any ants from the colony, we could manipulate individual-level access to information [22]. Using individual tagging to manipulate, the information available to certain animals is a powerful tool in understanding the mechanisms of collective decision-making [22,23]. We presented ant colonies (*Temnothorax albipennis*) with a choice between two new nests: one good and one poor. Each colony went through two treatments: in the ‘no-comparison’ treatment, ants which had visited the good nest were automatically prevented from visiting the poor nest, and vice versa; in the control treatment, all ants could visit both nests. Thus, in the ‘no-comparison’ treatment, no single individual had sufficient data to make a comparison, but the colony as a whole did have this information. In this way, we tested whether colonies could make successful decisions even when individuals were prevented from making direct comparisons between options, and we also explored the individual-level mechanisms through which effective collective choice can emerge.

## 2. Methods

Six *T. albipennis* colonies were collected from the Dorset Coast, UK, July–Oct 2009. Colonies were queenright and contained 70–150 workers and brood of all stages. Colonies were housed in artificial nests [15] and provided with water ad libitum and honey solution and *Drosophila melanogaster* weekly. During the experiment, each colony was subjected to two treatments: the ‘no-comparison’ treatment, and a control in which comparisons were possible. Of the two treatments, half the colonies experienced the control first and half the ‘no-comparison’ treatment. Trials using the same colony under different treatments were performed a week apart to eliminate the effects of experience from repeated emigrations [24]. This method provides paired control/treatment data at both the colony level and the level of individual radio-frequency identification (RFID)-tagged ants.

**Figure 1.**
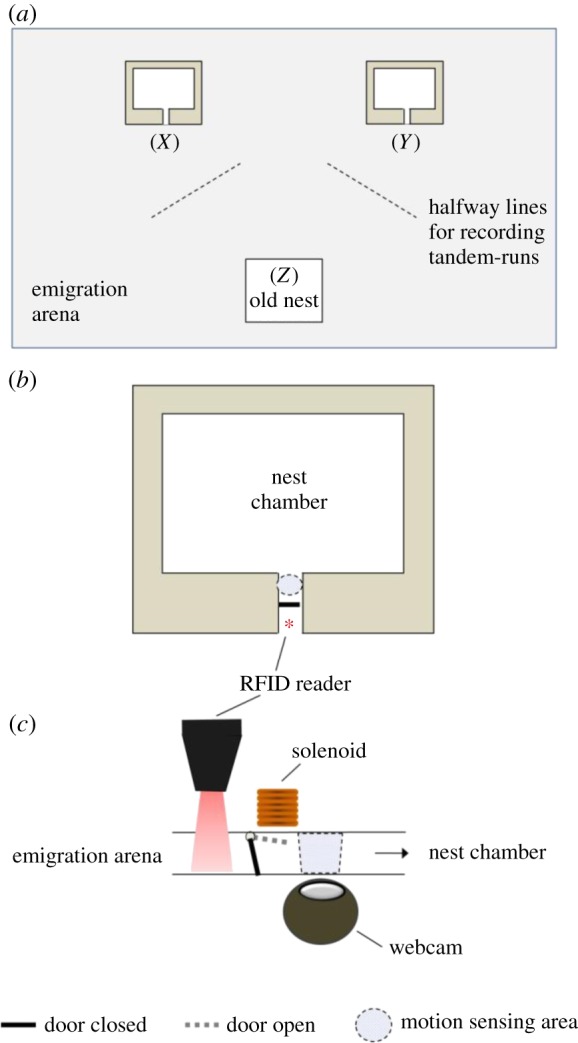
(*a*) Emigration arena. Distance from (*X*) to (*Y*) = 45 cm in the ‘nests far’ set-up; 10 cm in the ‘nests-near’ set-up. Distance (*X*) to (*Z*) = (*Y*) to (*Z*) = 32 cm in both set-ups. (*b*) New nest design with mechanism to control ants entering. (*c*) Side view of entrance corridor to nest showing mechanism of automatic doors, redrawn from [22]. (Online version in colour.)

The day before a colony's first trial, we tagged every worker ant in the colony with an RFID microtransponder (500 × 500 × 120 μm) with a unique identification (ID) [25,26]. The day before the colony's second trial, any ants which had lost their tags during the intertrial week (mean 22%) were retagged with new unique IDs. Trials were performed in a rectangular arena (75 × 43 cm) cleaned with water and alcohol between trials, with 7.5 cm high Fluon-coated walls (figure 1*a*). Two new nests with the same dimensions as the original nest were placed in the arena, each 32 cm from the old nest. The distance between the two new nests affects emigration dynamics, because when new nests are close together, colonies are likely to split, whereas when nests are far apart, fewer ants are likely to encounter both nests [15,16,20,27,28]. We therefore tested emigrations under two set-ups with different distances between the new nests. In the ‘nests far apart’ set-up, their entrances were 45 cm apart; in the ‘nests near’ set-up, their entrances were 10 cm apart. Three colonies were tested under the ‘nests far apart’ design which has the advantage that colonies are able to make clear unanimous choices between nests, but the drawback that relatively few ants actually visit both nests even in the control; three colonies were tested under the ‘nests near’ design, which increases the opportunity for ants to visit both nests, but results in more split decisions in which brood is transported into both nests. In both set-ups, one of the two new nests had a red filter covering the cavity area, to make the nest appear relatively dark to the ants [29]. Dark nests are more attractive to *T. albipennis*, so this nest is referred to as the ‘good nest’ [30]. The left–right positions of the good and poor nests were alternated between trials, and in addition, half the colonies had the good nest in the same position in the treatment and control, and half in the opposite position. Each new nest had an RFID reader (PharmaSeq, Inc., NJ) placed vertically over the entrance (figure 1*b,c*). When an RFID tag was detected, a LabView program initiated a digital output, which resulted in a current passing through a solenoid. This opened a small metal door magnetically, allowing the ant into the nest [22]. This process was so quick as to appear instantaneous. After 7 s, the solenoid switched off, and the door closed under gravity; this time interval allows a single ant through but prevents other ants being able to follow it [22]. This includes ants in a tandem run: the follower ant is unable immediately to follow its leader ant into the nest. An ant which had passed into the nest could exit again by triggering a motion-sensing webcam under the nest, which again caused the solenoid to open the door, this time for 6 s, as preliminary experiments showed that ants leave nests more quickly than they enter them. In the control trials, any RFID-tagged ant could enter either nest. In the ‘no-comparison’ treatment, any ant which entered nest A, was automatically placed on a ‘forbidden list’ for nest B, so that it could not trigger the door to nest B to open. Conversely, ants entering nest B could not subsequently enter nest A. This prevented any individual ant from entering both nests and being in a position to compare their qualities. This was maintained until the colony reached quorum in one of the nests [14], which was identified by the commencement of nest-mate carrying. The colony then fully migrated into the chosen nest. At quorum, the ‘forbidden lists’ were turned off, so any ant could enter either nest.

A trial began with the original nest-box placed in the centre of the arena and then destroyed by removing the lid. In addition to the RFID readers fixed over the entrances to the new nests, handheld RFID readers were used to read the tags on tandem-running ants as they crossed half-way lines (figure 1*a*). The identity of the leader and follower in the tandem run was recorded, and the direction of the tandem run was noted. This procedure does not disrupt tandem runs [20]. The time at which the transport of a nest-mate or a brood item first occurred to each nest was recorded. Emigrations were observed until the destroyed nest was completely empty of brood: at that point, the ‘initial choice’ of the colony was recorded, by counting the number of workers and brood items in each nest and recording the location of the queen. RFID readers were left in place recording activity at the nest entrances for 24 h, after which the ‘final choice’ of the colony was recorded in the same way. Use of the same colonies under both treatments allowed for paired analysis methods and collection of data on the same individual ants under different conditions. Data were collected from a total of 598 individual ants.

### (a) Statistical analysis

Statistical tests were performed in R version 3.0.1. Data were log-transformed for normality where necessary. To analyse the effect of treatment on the nest-visiting behaviour of scouting ants, linear-mixed-effect models were used (R package nlme). For model 1, the dependent variable was number of unique ants visiting nest per hour. For model 2, the dependent variable was number of visits per ant per hour. Dependent variables were weighted by the total number of active ants for which data were available. Both maximal models included fixed factors: treatment (‘no-comparison’/control), nests visited (good only/poor only/both), set-up (nests near/nests far) and two-way interactions. Colony was included as a random factor, because each colony was subjected to both treatments. Minimal models (electronic supplementary material, table S1) were found by sequential removal of non-significant terms, testing for improvements in model fit using ANOVA. For analysis of switching between nests, only data from the ‘nests near’ set-up is used, because very few ants discover both nests in the nests far set-up (table 1). To analyse whether general activity levels predict whether an ant engages in switching between nests, a generalized linear-mixed model (GLMM) was used (R package MASS). Switching between nests was the binary-dependent variable, with activity (measured as total number of nest visits pre-quorum) as a fixed factor, ant identity as a random factor and a binomial error structure.

**Table 1.** Choice outcomes for colonies making decisions under each treatment (‘no-comparison’ and control). (Total number of ants in the colony, number of ants that were ‘active’, i.e. made at least two nest visits during the decision-making period and number of ants which either attempted (‘no-comparison’ treatment) or succeeded (control treatment) in visiting both nests.)

colony	set-up	no. ants in colony	‘no-comparison’ choice outcome	no. active ants	no. ants attempt to switch	control choice outcome	no. active ants	no. ants switch
A	nests far	142	good nest	28	6	good nest	16	3
B	nests far	86	good nest	12	1	good nest	12	1
C	nests far	91	good nest	11	1	good nest	16	1
D	nests near	70	good nest	27	12	split	20	5
E	nests near	130	split	32	8	split	25	1
F	nests near	114	split	37	11	split	26	2
total		633		147	39		115	13

## 3. Results

### (a) Colony-level decision-making

Colonies under the ‘no-comparison’ treatment successfully chose the good nest when the two new nests were distant from each other, but tended to split between the nests when they were placed close together (table 1). The paired control data show similar patterns, with most colonies performing at the same level of accuracy whether allowed to make comparisons or not, and a single colony actually performing worse in the control than the ‘no-comparison’ treatment. In general, it is clear that preventing comparisons does not itself prevent effective decision-making and in terms of decision-accuracy the ‘no-comparison’ treatment did no worse than the control. Speed of decision-making (taken as time until nest-mate transport begins) was very similar between the two treatments (no-comparison: median = 38 min, range = 25–175; control: median = 38 min, range = 21–107; paired *t*-test, *t*_5_ = 0.97, *p* = 0.38), indicating that the ‘no-comparison’ treatment does not cause colonies to choose more slowly. The increased splitting when the nests were close together is likely to have been owing to ants making errors during the recruitment process, contributed to by the disruption to tandem runs caused by the door mechanism in both the ‘no-comparison’ and the control treatments. The doors forced the follower to wait 7 s before entering the nest. When the new nests were far apart, most followers (86%, *n* = 14) subsequently entered the nest to which they were recruited, whereas when the nests were close together, only 30% (*n* = 10) of followers entered the nest to which they were recruited, with the majority entering the other nest. This significant difference (Fisher's exact test, *p* < 0.02) suggests that during the delay caused by the doors, the followers search locally, and when there is another nest nearby, enter that nest instead.

### (b) Individual-level decision-making

**Figure 2.**
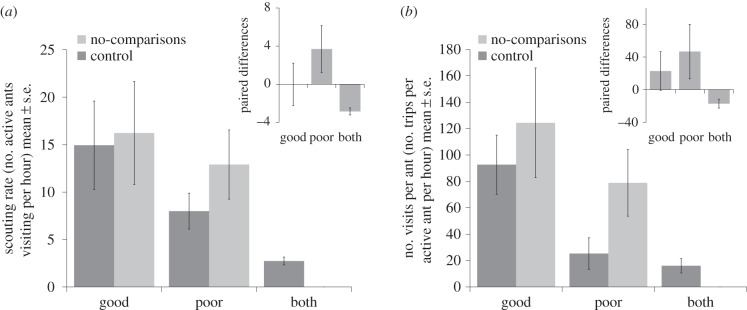
Ant activity during the decision-making period. (*a*) Number of unique ants visiting either only one of the two new nests or both nests. (*b*) Number of visits per ant involved in visiting one or both of the nests. Distributions of ants and visits per ant differ significantly between the ‘no-comparison’ treatment and the control (electronic supplementary material, table S2). Paired data from the same colonies undergoing both treatments are used in the analysis: insets show the paired differences in scouting behaviour, i.e. for each colony, the ‘no-comparison’ treatment minus the control treatment.

The behaviour of the nest-scouting ants differed between the ‘no-comparison’ treatment and the control (figure 2 and the electronic supplementary material, table S1). Similar numbers of ants were involved in visiting the good nest only across both treatments (figure 2*a*; lme: *t*_25_ = 1.49, n.s.), whereas ants visiting only the poor nest were rarer in the control than in the ‘no-comparison’ treatment (lme: *t*_25_ = 4.01, *p* < 0.001). In the control, ants visiting the poor nest are able to switch to the other nest; hence, they have the opportunity to visit both nests, whereas, in the ‘no-comparison’ treatment, ants which visit the poor nest have only that nest available to them. The same pattern is seen with the number of nest visits per ant (figure 2*b* and the electronic supplementary material, table S1), with similar numbers of visits per ant to the good nest only (lme: *t*_25_ = 1.45, n.s.), but more visits per ant to the poor nest only in the ‘no-comparison’ treatment where the ants could not switch nest (lme: *t*_25_ = 4.76, *p* < 0.001).

The two nests are equally likely to be discovered first in both ‘no-comparison’ and control trials (*χ*_1_^2^ = 2.79, *p* = 0.1); however, overall, more ants visit the good nest during the decision-making period (lme: *t*_25_ = 5.62, *p* < 0.0001). This effect is mostly accounted for by the control trials (good nest: 15 ± 5 mean ants per hour ± s.e.; poor nest: 8 ± 2) and is likely to be owing to a higher probability of recruitment to the good nest. Although the doors disrupt tandem runs at the entrance to the nest even in the control treatment, the recruitment process will still bring naive ants to the vicinity of the nest. In the ‘no-comparison’ treatment (good nest: 16 ± 5 mean ants per hour ± s.e.; poor nest 12 ± 4), some of these ants will be on the ‘forbidden list’, so will be unable to enter the good nest to which they have been recruited.

How does being denied entry affect the ants' behaviour? We have RFID data on both the ants that switch between nests in the control, and on the ants that attempted to switch but were denied entry in the ‘no-comparison’ treatment. Similar numbers of ants attempt to switch between nests across the two treatments (control: 24% ±15 of active ants per hour; ‘no comparison’ treatment 30% ±20 of active ants per hour; paired *t*-test: *t*_5_ = 0.66, *p* = 0.54), however, ants make more attempted switches back and forth between the nests in the ‘no-comparison’ treatment than real switches in the control (‘no-comparison’: range = 0–4; control: range = 0–2, Kruskall–Wallis test: *H*_1_ = 10.44, *p* = 0.001). Taking, more specifically, the ants most affected by the doors, i.e. those who have found the poor nest and then (in the ‘no-comparison’ treatment) are prevented from entering the good nest (figure 2*a*), we find that these ants make on average 4.3 ± 2.5 (mean ± s.d.) subsequent visits per attempted visit. By contrast, ants in the control treatment that go from the poor to the good nest are likely to then stay at the good nest, showing lower levels of subsequent activity (control 2.3 ± 1.3 subsequent visits; *t*-test: *t*_9_ = 2.35, *p* < 0.05). This shows that the ants experiencing the poor nest continue searching for longer when there is no other option accessible to them.

### (c) Consistent differences in individual thresholds?

Ants engaging in one emigration are disproportionately more likely to engage in the second emigration than other still-tagged ants (log-linear analysis across the six colonies, *G*^2^_1_ = 53.9, *p* < 0.0001). Taking the 207 individual ants for which we have data on participation in two emigrations, there is a positive correlation between activity in one emigration and the next (*ρ* = 0.44, *n* = 206, *p* < 0.001; electronic supplementary material, figure S1). Overall scouting activity does not differ between first and second emigrations (Wilcoxon signed-rank test, *V* = 9294, *n* = 207, *p* = 0.84) or between ‘no-comparison’ and control treatments (Wilcoxon signed-rank test, *V* = 9582, *n* = 207, *p* = 0.87).

Ants that switch between the two nests (or attempt to do so, in the ‘no-comparison’ treatment) in their first emigration are disproportionately likely to do this again in their second emigration (*χ*_1_^2^ = 4.932, *p* < 0.05; table 2). Overall activity does not significantly predict switching within this group (ant ID is taken into account as a random factor; GLMM: *t*_37_ = 1.64, *p* = 0.11), so the behaviour of these persistent switchers cannot simply be explained by them being highly active ants.

**Table 2.** Consistency in switching behaviour across the two emigrations. (Ants are included only if they retained their tags for both emigrations and in both of the emigrations made at least two nest visits before quorum was reached, and therefore could feasibly have switched between nests in both emigrations (*n* = 38).)

	ants switched between nests:			
	both emigrations	first emigration only	second emigration only	neither emigration
no. ants	6	5	5	22
chi-squared contribution	2.49	1.01	1.01	0.41
test results:	χ^2^_1_ = 4.93, *p* < 0.05			

## 4. Discussion

Our results clearly show that preventing individual comparisons does not adversely affect accuracy or speed of collective decision-making in house-hunting ants, relative to paired controls in which individual comparisons were permitted. This is consistent with a threshold rule of decision-making in which ants make decisions about whether or not to commit to a nest based only on the quality of that nest, relative to an internal standard and not in relation to other nests [21]. These results are also consistent with the behaviour of pheromone trail-laying ant colonies that are able to collectively choose between differing options: the shorter of two paths or the better of two food sources. In these cases, the collective decisions emerge from the interactions of foragers mediated via the pheromone trail, and can be explained by models which do not invoke individual-level comparison [31–34]. Individual access to information has not been directly manipulated in this foraging system, but has in honeybee swarms choosing between new nest-boxes [35]. Removing bees that encountered both of the nest-boxes (about 18% of scouts) did not prevent or delay swarms from choosing a nest [35]; however, the boxes were equidistant and of equal quality, so comparison might be expected to be of limited importance. Our results show that even when options differ in attractiveness, preventing comparisons does not impair collective choice.

At the individual-level, our results show that preventing scouts from having access to more than one nest does affect the behaviour of the ants during the decision-making process. In the ‘no-comparison’ treatment, ants that have visited the poor nest, then are recorded attempting (and failing) to enter the good nest, are subsequently more active at the entrances to both nests than in the control treatment, in which they would be able actually to enter the good nest. This behaviour matches what would be predicted by a simple threshold model of decision-making [21], in which ants that reject a poor nest search for alternatives.

The individual-level data also suggest why preventing individual comparisons does not affect colony-level decision-making: both the number of ants visiting the good nest and the number of visits per ant to the good nest are similar between the treatments; it is the behaviour of the ants visiting the poor nest first that is most affected by our manipulation. So, in both treatments, it is possible for the colony to reach quorum (i.e. a number of ants sufficient to trigger nest-mate carrying behaviour and full emigration) in the good nest, even though in the ‘no-comparison’ treatment, the ants that first visit the poor nest cannot switch to the good nest and contribute to this quorum. This smaller number of available ants could be expected to slow down the process of reaching quorum in the ‘no-comparison’ treatment [28], but we do not see a significant difference in speed of decision-making. This may be because we are providing the colonies with a very simple choice challenge. We know that *T. albipennis* colonies can solve more complex problems and work over much greater scales [15,36]. In our experiment, the environment is very simple visually and the ants find the two nests easily, so the scouting population is not widely dispersed across many possible sites. This may mean that the time taken to make decisions in the simple context is close to the minimum possible decision time, and making a few extra scouts available therefore makes no appreciable difference to the speed of achieving quorum. This idea is supported by the increased level of splitting between the two new nests that was seen when the nests were brought closer together. This made the discovery of both nests easier, both because they may have combined to make an area of visual interest, and because of the slightly disrupted recruitment caused by the doors on the nests. These doors let only one ant enter at once, so even in the control treatment the following ant would have to wait a little, and was likely to discover the other nearby nest instead. House-hunting ants are subjected to a speed-cohesion trade-off, and rapid discovery of both nests by many individuals means that it is possible for both to reach quorum quickly and around the same time whereupon the colony will split.

Given that individual ants do have the ability to make comparative assessments [17], why does this capacity seem to play no role in colony decisions? Would these ants in their natural habitat even have the opportunity to visit more than one nest, and therefore be in a position to make comparisons? There have been no field studies that address this directly, however, inter-nest distances in the wild can be low (frequently less than 30 cm; E. J. H. Robinson and N. R. Franks 1992–2013, personal observation) compared with the much greater distances over which laboratory colonies will readily emigrate, with scouts visiting pairs of nests that are separated by as much as 1.2 m [20,37]. This gives an indication that we would expect some ants to have the opportunity to encounter multiple sites in the wild, however, across the scouting population, the information obtained may be very variable. Our findings illustrate that the collective decision process is robust to differences in individual access to information and the resulting behavioural changes. This may be important in a more natural, heterogeneous and unpredictable environment, where decisions may need to be made so quickly that there is no time to rely on individuals interrogating a wide set of options. Even in the relatively simple environment of our experiments, when we placed the new nests far apart, few ants encountered both the nests before the colony reached quorum in one and began transport to that nest. The time costs and diminishing returns of collecting more information mean animals may benefit from truncating the information gathering process and making a quicker and sufficiently accurate (rather than maximally accurate) decision [38,39].

The ability of individual ants to make comparative assessments might play a more subtle role in colony organization. The emergency emigrations investigated here, analogous to the breaking open of the fragile rock cavities in which these ants nest, are likely to be driven by the necessity for speed. Ant colonies also emigrate simply to improve their nest conditions, while their old nest is still intact [40]. In this context, speed is much less critical, and comparative assessment might potentially have a role to play. For example, the ability to make comparisons could be used to update an ant's individual acceptance threshold very slowly, so that ants which experience only poor nests gradually reduce their threshold, thus adjusting to their environment. Some adjustment to the local environment is possible without threshold change, if there is a distribution of acceptance thresholds within the colony. In this case, colonies housed in a poor nest would have a larger proportion of dissatisfied ants who would be likely to search for a better option, whereas well-housed colonies would have few ants inclined to search [41,42]. Updating acceptance thresholds could add a level of fine-tuning to this: further investigation of the role of comparative assessment in other decision contexts is required.

The existence of a distribution of acceptance thresholds remains a hypothesis [20,21,42], but the individual-level data from this experiment do shed some light on this area. Some individual ants were recorded as playing an active role in both of the emigrations performed by their colony. In general, individual activity levels were correlated across the two emigrations. Variation in activity level among ants is well known [25], but interestingly, in this experiment ants that switched between nests in one emigration were likely to switch in the other emigration and this could not be simply explained by high activity levels in these ants. The results are consistent with these ants being ‘high threshold ants’ which switch (or attempt to, in the ‘no-comparison’ treatment), because the nest they have encountered does not meet their acceptance threshold. A colony's threshold distribution would be predicted to influence its ability to respond to decision-making challenges and is an interesting subject for future study.

By manipulating the access of individuals to parts of the information set, we have shown that collective comparisons can emerge from the interactions of poorly informed individuals. In this example, the ant colony cannot simply ‘copy’ a good decision made by a single ant, but must reach a conclusion that is evident only on the collective scale. Collective decision-makers can thus enjoy all the benefits of comparison, in terms of taking the full choice set into consideration, but without potential drawbacks such as irrational behaviours and cognitive overload.

## Supplementary Material

Electronic Supplementary Information

## References

[1] Sueur C Deneubourg JL Petit O 2010 Sequence of quorums during collective decision making in macaques. Behav. Ecol. Sociobiol. 64, 1875–1885. (10.1007/s00265-010-0999-8)

[2] Vonk J Beran MJ 2012 Bears ‘count’ too: quantity estimation and comparison in black bears, *Ursus americanus*. Anim. Behav. 84, 231–238. (10.1016/j.anbehav.2012.05.001). 22822244 PMC3398692

[3] Svensson BG Petersson E Forsgren E 1989 Why do males of the dance fly *Empis borealis* refuse to mate? The importance of female age and size. J. Insect Behav. 2, 387–395. (10.1007/BF01068063)

[4] Uy JAC Patricelli GL Borgia G 2001 Complex mate searching in the satin bowerbird *Ptilonorhynchus violaceus*. Am. Nat. 158, 530–542. (10.1086/323118) 18707307

[5] Dowds BM Elwood RW 1983 Shell wars: assessment strategies and the timing of decisions in hermit crab shell fights. Behaviour 85, 1–24. (10.1163/156853983X00011)

[6] Doyle JR O'Connor DJ Reynolds GM Bottomley PA 1999 The robustness of the asymmetrically dominated effect: buying frames, phantom alternatives, and in-store purchases. Psychol. Mark. 16, 225–243. (10.1002/(SICI)1520-6793(199905)16:3<225::AID-MAR3>3.0.CO;2-X)

[7] Bateson M 2004 Mechanisms of decision-making and the interpretation of choice tests. Anim. Welfare 13, 115–120.

[8] Latty T Beekman M 2011 Irrational decision-making in an amoeboid organism: transitivity and context-dependent preferences. Proc. R. Soc. B 278, 307–312. (10.1098/rspb.2010.1045) PMC3013386 20702460

[9] Hensher DA 2006 How do respondents process stated choice experiments? Attribute consideration under varying information load. J. Appl. Econ. 21, 861–878. (10.2307/25146470).

[10] Langen TA 1999 How western scrub-jays (*Aphelocoma californica*) select a nut: effects of the number of options, variation in nut size, and social competition among foragers. Anim. Cogn. 2, 223–233. (10.1007/s100710050043)

[11] Raffa KF Havill NP Nordheim EV 2002 How many choices can your test animal compare effectively? Evaluating a critical assumption of behavioral preference tests. Oecologia 133, 422–429. (10.1007/s00442-002-1050-1). 28466207

[12] Seeley TD Visscher PK 2004 Group decision making in nest-site selection by honey bees. Apidologie 35, 101–116. (10.1051/apido:2004004)

[13] Seeley TD Buhrman SC 1999 Group decision making in swarms of honey bees. Behav. Ecol. Sociobiol. 45, 19–31. (10.1007/s002650050536)

[14] Pratt SC Mallon EB Sumpter DJT Franks NR 2002 Quorum sensing, recruitment, and collective decision-making during colony emigration by the ant *Leptothorax albipennis*. Behav. Ecol. Sociobiol. 52, 117–127. (10.1007/s00265-002-0487-x)

[15] Franks NR Hardcastle KA Collins S Smith FD Sullivan KME Robinson EJH Sendova-Franks AB 2008 Can ant colonies choose a far-and-away better nest over an in-the-way poor one? Anim. Behav. 76, 323–334. (10.1016/j.anbehav.2008.02.009)

[16] Franks NR Mallon EB Bray HE Hamilton MJ Mischler TC 2003 Strategies for choosing between alternatives with different attributes: exemplified by house-hunting ants. Anim. Behav. 65, 215–223. (10.1006/anbe.2002.2032)

[17] Edwards S Pratt SC 2009 Rationality in collective decision-making by ant colonies. Proc. R. Soc. B 276, 3655–3661. (10.1098/rspb.2009.0981) PMC2817311 19625319

[18] Sasaki T Pratt SC 2012 Groups have a larger cognitive capacity than individuals. Curr. Biol. 22, R827–R829. (10.1016/j.cub.2012.07.058) 23058797

[19] Sasaki T Pratt SC 2011 Emergence of group rationality from irrational individuals. Behav. Ecol. 22, 276–281. (10.1093/beheco/arq198)

[20] Robinson EJH Smith FD Sullivan KME Franks NR 2009 Do ants make direct comparisons? Proc. R. Soc. B 276, 2635–2641. (10.1098/rspb.2009.0350). PMC2686663 19386652

[21] Robinson EJH Franks NR Ellis S Okuda S Marshall JAR 2011 A simple threshold rule is sufficient to explain sophisticated collective decision-making. PLoS ONE 6, e19981 (10.1371/journal.pone.0019981) 21629645 PMC3101226

[22] Robinson EJH Feinerman O Franks NR 2012 Experience, corpulence and decision-making in ant foraging. J. Exp. Biol. 215, 2653–2659. (10.1242/jeb.071076) 22786642

[23] Fleischmann D 2013 Female Bechsteins bats adjust their group decisions about communal roosts to the level of conflict of interests. Curr. Biol. 23, 1658–1662. (10.1016/j.cub.2013.06.059) 23954425

[24] Langridge EA Sendova-Franks AB Franks NR 2008 The behaviour of ant transporters at the old and new nests during successive colony emigrations. Behav. Ecol. Sociobiol. 62, 1851–1861. (10.1007/s00265-008-0614-4)

[25] Robinson EJH Richardson TO Sendova-Franks AB Feinerman O Franks NR 2009 Radio-tagging reveals the roles of corpulence, experience and social information in ant decision making. Behav. Ecol. Sociobiol. 63, 627–636. (10.1007/s00265-008-0696-z)

[26] Robinson EJH Feinerman O Franks NR 2009 Flexible task allocation and the organisation of work in ants. Proc. R. Soc. B 276, 4373–4380. (10.1098/rspb.2009.1244) PMC2817103 19776072

[27] Mallon EB Pratt SC Franks NR 2001 Individual and collective decision-making during nest site selection by the ant *Leptothorax albipennis*. Behav. Ecol. Sociobiol. 50, 352–359. (10.1007/s002650100377)

[28] Seeley TD Visscher PK 2004 Quorum sensing during nest-site selection by honeybee swarms. Behav. Ecol. Sociobiol. 56, 594–601. (10.1007/s00265-004-0814-5)

[29] Briscoe AD Chittka L 2001 The evolution of color vision in insects. Annu. Rev. Entomol. 46, 471–510. (10.1146/annurev.ento.46.1.471) 11112177

[30] Franks NR Dornhaus A Metherell B Nelson T Lanfear SA Symes W 2006 Not everything that counts can be counted: ants use multiple metrics for a single nest trait. Proc. R. Soc. B 273, 165–169. (10.1098/rspb.2005.3312). PMC1560019 16555783

[31] Beckers R Deneubourg JL Goss S 1992 Trails and U-turns in the selection of a path by the ant *Lasius niger*. J. Theor. Biol. 159, 397–415. (10.1016/S0022-5193(05)80686-1)

[32] Goss S Deneubourg JL Pasteels JM 1989 Self-organized shortcuts in the Argentine ant. Naturwissenschaften 76, 579–581. (10.1007/BF00462870)

[33] Pasteels JM Deneubourg JL Verhaeghe JC Boeve JL Quinet Y 1986 Orientation along terrestrial trails by ants. In Mechanisms in insect olfaction (eds Payne TL Birch MC Kennedy CEJ), pp. 131–138. Oxford, UK: Clarendon Press.

[34] Beckers R Deneubourg JL Goss S 1993 Modulation of trail laying in the ant *Lasius niger* (Hymenoptera, Formicidae) and its role in the collective selection of a food source. J. Insect Behav. 6, 751–759. (10.1007/BF01201674)

[35] Visscher PK Camazine S 1999 Collective decisions and cognition in bees. Nature 397, 400 (10.1038/17047) 29667972

[36] Franks NR Richardson TO Stroeymeyt N Kirbya RW Amosa WMD Hogan PM Marshall JAR Schlegel T 2013 Speed-cohesion trade-offs in collective decision making in ants and the concept of precision in animal behaviour. Anim. Behav. 85, 1233–1244. (10.1016/j.anbehav.2013.03.010)

[37] Basari N Bruendl AC Hemingway CE Roberts NW Sendova-Franks AB Franks NR 2014 Landmarks and ant search strategies after interrupted tandem runs. J. Exp. Biol. 217, 944–954. (10.1242/jeb.087296) 24198259

[38] Rinberg D Koulakov A Gelperin A 2006 Speed–accuracy tradeoff in olfaction. Neuron 51, 351–358. (10.1016/j.neuron.2006.07.013) 16880129

[39] Chittka L Skorupski P Raine NE 2009 Speed-accuracy tradeoffs in animal decision making. Trends Ecol. Evol. 24, 400–407. (10.1016/j.tree.2009.02.010) 19409649

[40] Dornhaus A Franks NR Hawkins RM Shere HNS 2004 Ants move to improve: colonies of *Leptothorax albipennis* emigrate whenever they find a superior nest site. Anim. Behav. 67, 959–963. (10.1016/j.anbehav.2003.09.004)

[41] Doran C Pearce T Connor A Schlegel T Franklin EL Sendova-Franks AB Franks NR 2013 Economic investment by ant colonies in searches for better homes. Biol. Lett. 9, 20130685 (10.1098/rsbl.2013.0685) 24088565 PMC3971717

[42] Stroeymeyt N Robinson EJH Hogan PM Marshall JAR Giurfa M Franks NR 2011 Experience-dependent flexibility in collective decision-making by house-hunting ants. Behav. Ecol. 22, 535–542. (10.1093/beheco/arr007)

