# Supplementary material for: How collective comparisons emerge without individual comparisons of the options
Source: Proc Biol Sci. 2014 Jul 22;281(1787):20140737. doi: 10.1098/rspb.2014.0737 (PMC4071554; doi:10.1098/rspb.2014.0737)
Supplement: Electronic Supplementary Information [file rspb20140737supp1.pdf]

Electronic Supplementary Material

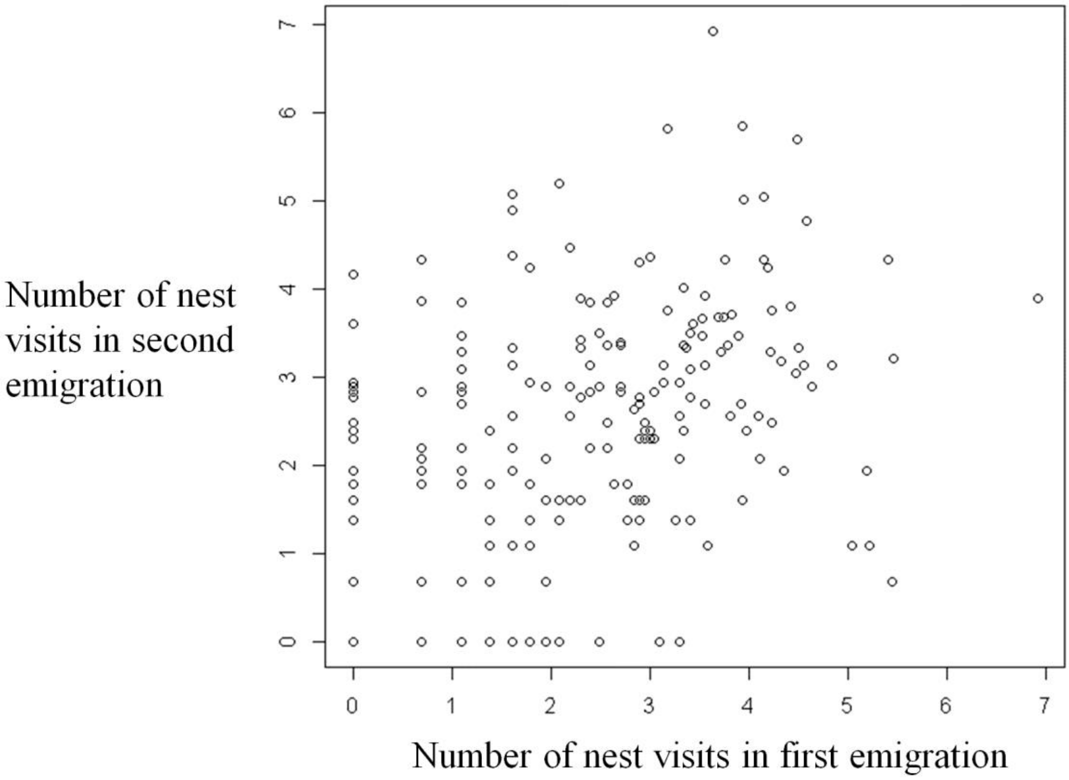

Figure S1. Correlation between level of activity in the first and second emigrations. Both axes are logged for presentation (Spearman's rho=0.44, n=206, p<0.001)

Model 1. Number of unique ants visiting nests during decision-making	t-value	df	p
Treatment	2.23	25	<0.04*
Nest (Good)	6.09	25	<0.0001***
Nest (Poor)	9.26	25	<0.0001***
Treatment*Nest(Good)	1.49	25	0.145
Treatment*Nest(Poor)	4.01	25	<0.001**
Model 2: Numbers of visits per ant during decision-making	t-value	df	p
Treatment	2.32	25	<0.02*
Nest (Good)	6.61	25	<0.0001***
Nest (Poor)	8.28	25	<0.0001***
Setup	3.65	4	<0.03*
Treatment*Nest(Good)	1.45	25	0.160
Treatment*Nest(Poor)	4.76	25	<0.001**

Table S1. Minimal models showing effect of treatment on scouting behaviour of ants during decision-making.*p<0.05; **p<0.001; ***p<0.0001

Colony	Treatment first	Number of ants in colony	Number of recorded ants retagged between trials[1]	Number of ants recorded at some point during both emigrations	Ants seen in both treatments, as % of number possible[2]
A	No comparison	142	9 (24)	34	66.7%
B	No comparison	86	16 (43)	23	82.1%
C	Control	91	4 (13)	26	78.8%
D	Control	70	7 (16)	24	88.9%
E	Control	130	6 (23)	56	77.8%
F	No comparison	114	7 (23)	44	83.0%

Table S2. Re-recording of tagged ants in repeated emigrations.

[1] Some ants lost their tags in between the first and second treatment, so we re-tagged before the second began. Number in brackets is the total re-tagged, whether recorded in the emigration or not.

[2] Ants which were re-tagged could not be recorded in both treatments, so this column shows ants which were actually recorded in both treatments as a proportion of ants which could potentially have been recorded in both treatments because they were recorded in either one of the treatments and were not-retagged.

Numbers of visits in the pre-quorum period of emigrations:

Colony	ID	First emigration total	Second emigration total
A	A_8	3	2
A	A_94	13	47
A	A_118	2	2
A	A_123	2	77
A	A_139	1	1
A	A_174	1	1
A	A_186	1	2
A	A_216	5	80
A	A_218	1	4
A	A_260	1	5
A	A_270	2	48
A	A_308	3	7
A	A_327	9	18
A	A_370	1	19
A	A_388	3	18
A	A_396	2	9
A	A_455	4	6
A	A_462	35	51
A	A_469	8	1
A	A_521	1	1
A	A_558	1	6
A	A_609	20	79
A	A_610	3	1
A	A_615	1	65
A	A_634	1	1
A	A_706	2	1
A	A_750	10	31
A	A_764	43	76
A	A_804	1	2
A	A_857	1	4
A	A_866	4	3
A	A_943	1	4
A	A_977	5	134
A	A_1011	1	1
B	B_32	1	1
B	B_48	87	21
B	B_114	5	7
B	B_233	13	12
B	B_320	2	1
B	B_353	222	77
B	B_426	14	51
B	B_533	20	10
B	B_590	1003	49
B	B_631	1	2
B	B_654	15	17
B	B_703	41	27
B	B_731	8	180
B	B_761	28	29

B	B_817	13	29
B	B_826	15	29
B	B_854	5	161
B	B_873	7	1
B	B_942	23	23
B	B_948	3	6
B	B_953	90	28
B	B_1004	5	9
B	B_1022	52	151
C	C_28	19	10
C	C_50	29	28
C	C_55	3	2
C	C_60	69	43
C	C_93	97	119
C	C_97	3	22
C	C_106	35	23
C	C_202	75	24
C	C_238	6	19
C	C_247	1	2
C	C_280	1	1
C	C_390	5	13
C	C_414	1	10
C	C_477	230	2
C	C_500	11	47
C	C_564	155	3
C	C_594	31	37
C	C_596	11	17
C	C_614	1	12
C	C_627	5	1
C	C_752	4	2
C	C_816	1	2
C	C_824	20	11
C	C_914	6	1
C	C_924	9	88
C	C_998	7	5
D	D_41	63	77
D	D_66	30	4
D	D_70	12	1
D	D_101	7	8
D	D_156	12	18
D	D_203	66	70
D	D_205	11	9
D	D_388	26	4
D	D_446	1	37
D	D_599	50	15
D	D_603	3	9
D	D_705	30	22
D	D_792	12	33
D	D_793	30	16
D	D_797	17	14

D	D_830	34	32
D	D_871	11	23
D	D_880	1	16
D	D_884	89	301
D	D_929	13	9
D	D_934	18	15
D	D_946	1	11
D	D_973	1	18
D	D_978	6	70
E	E_7	51	349
E	E_24	2	6
E	E_57	2	8
E	E_82	10	49
E	E_90	15	30
E	E_92	233	25
E	E_100	19	5
E	E_110	21	10
E	E_113	184	3
E	E_119	68	27
E	E_123	18	10
E	E_146	4	4
E	E_182	3	17
E	E_184	23	19
E	E_206	24	43
E	E_209	36	3
E	E_257	3	15
E	E_279	18	5
E	E_295	10	5
E	E_317	3	32
E	E_321	8	5
E	E_342	49	32
E	E_352	27	13
E	E_379	53	11
E	E_401	18	16
E	E_453	3	27
E	E_490	27	8
E	E_503	6	6
E	E_518	61	8
E	E_528	1	2
E	E_563	17	5
E	E_569	19	12
E	E_608	38	1027
E	E_622	6	3
E	E_626	9	5
E	E_667	2	7
E	E_687	15	18
E	E_694	2	1
E	E_695	10	16
E	E_708	2	1
E	E_713	34	39

E	E_742	16	4
E	E_776	7	18
E	E_787	16	6
E	E_839	77	7
E	E_841	6	4
E	E_844	2	17
E	E_856	1	17
E	E_883	1	4
E	E_889	1	6
E	E_939	28	11
E	E_940	24	335
E	E_949	5	28
E	E_952	95	23
E	E_972	5	3
E	E_985	4	11
F	F_72	103	18
F	F_118	69	12
F	F_186	45	13
F	F_193	178	7
F	F_207	6	3
F	F_282	4	1
F	F_283	63	156
F	F_304	27	19
F	F_307	51	5
F	F_336	22	1
F	F_359	5	1
F	F_404	10	28
F	F_426	44	29
F	F_436	2	1
F	F_449	18	4
F	F_466	4	1
F	F_506	4	1
F	F_512	8	4
F	F_524	42	40
F	F_525	1	1
F	F_545	21	17
F	F_553	5	23
F	F_558	1	7
F	F_559	83	45
F	F_590	46	41
F	F_644	30	33
F	F_653	35	15
F	F_655	2	1
F	F_673	2	1
F	F_677	27	1
F	F_682	14	6
F	F_715	126	23
F	F_718	17	3
F	F_727	18	74
F	F_731	40	40

F	F_763	17	3
F	F_785	16	6
F	F_840	9	13
F	F_846	60	13
F	F_894	7	2
F	F_896	28	56
F	F_901	19	11
F	F_910	3	47
F	F_912	3	1

Treatment	Colony	Nest	Number of ants per hour	Number of visits per ant per hour	Set-up	Weighting by activity
Control	A	Good	4.44	53.61	Far	14
Control	A	Poor	8.89	88.06	Far	14
Control	A	Both	2.29	4.32	Far	14
Control	B	Good	11.43	47.86	Far	6
Control	B	Poor	2.86	5.71	Far	6
Control	B	Both	4.40	8.79	Far	6
Control	C	Good	5.05	32.40	Far	14
Control	C	Poor	2.24	14.44	Far	14
Control	C	Both	1.05	3.15	Far	14
Control	D	Good	6.00	90.00	Near	14
Control	D	Poor	4.00	8.91	Near	14
Control	D	Both	4.00	40.00	Near	14
Control	E	Good	24.55	95.15	Near	14
Control	E	Poor	10.91	11.68	Near	14
Control	E	Both	2.73	10.91	Near	14
Control	F	Good	31.43	184.94	Near	18
Control	F	Poor	14.29	14.70	Near	18
Control	F	Both	2.86	22.86	Near	18
No comparison	A	Good	3.09	21.50	Far	23
No comparison	A	Poor	4.80	4.09	Far	23
No comparison	A	Both	0.00	0.00	Far	23
No comparison	B	Good	9.23	68.97	Far	7
No comparison	B	Poor	1.54	1.54	Far	7
No comparison	B	Both	0.00	0.00	Far	7
No comparison	C	Good	9.38	53.91	Far	6
No comparison	C	Poor	5.63	6.88	Far	6
No comparison	C	Both	0.00	0.00	Far	6
No comparison	D	Good	6.72	35.69	Near	24
No comparison	D	Poor	4.66	77.59	Near	24
No comparison	D	Both	0.00	0.00	Near	24
No comparison	E	Good	16.22	155.68	Near	29
No comparison	E	Poor	24.32	170.27	Near	29
No comparison	E	Both	0.00	0.00	Near	29
No comparison	F	Good	38.40	281.44	Near	28
No comparison	F	Poor	19.20	81.60	Near	28
No comparison	F	Both	0.00	0.00	Near	28
